# Observations on the Sounds of the Heart

**Published:** 1852-02

**Authors:** Richard Brown

**Affiliations:** Cobham


*Observations on the Sounds of the Heart.* By RICHARD BROWN, Esq., M. R. C. S., L. A. C., &c., Cobham.—Few subjects, perhaps, have claimed more attention from physiologists than the one upon which I am about to offer some remarks, and I may add, none in which a greater discrepancy of opinion has been manifested.

Harvey and Haller describe the contraction of the auricles as preceding those of the ventricles. Læennec conceived that the contraction of the auricles followed those of the ventricles. Turner, Corrigan, Williams, Hope and Bouillaud, have shown the inaccuracy of Læennec's opinion. Dr. Williams investigated this subject, and his inferences were confirmed by the Committees of the British Association—namely, that the contraction of the ventricles followed immediately that of the auricles.

The first sound of the heart was ascribed by Mr. Carlisle to the rush of blood into the great arteries; by Mons. Rouanct and others to the closing of the ariculo-ventricular valves; by Dr. Hope, to the collision of the particles of fluid in the ventricles; and by Dr. Williams, to the muscular contraction of the heart itself.

The second sound was ascribed by Dr. Hope to the impulse of the blood from the auricles filling the ventricles; by Messrs. Carlile, Carswell, Rouanet, Bouillaud, and others, to the suction of the ventricles, causing the elevation of the sigmoid valves, and to the reaction of the arterial columns of blood against these valves.

The experiments performed by Dr. Williams, assisted by Dr. Hope and others, in order to determine these points, proved that the first sound is produced by the muscular contraction of the ventricles, and that the second sound is caused by the reaction of the arterial columns of blood, tightening the semilunar valves at the diastole of the ventricles.

The first motion of the heart following the interval of repose is the systole of the auricle, which I consider accompanies the diastole of the ventricle; and the systole of the ventricle immediately follows its diastole, commences suddenly, and considerably diminishes the volume of the organ.

On applying the ear or a stethoscope over the præcordial region, two sounds are heard following each other; the first is dull and prolonged, whilst the second is shorter and sharper. The first sound is produced during the diastole, and the second during the systole of the ventricles; and in support of this theory I will briefly state the circumstances under which this opinion was formed.

Some few weeks since, attending a patient laboring under increased action of the heart, and whilst conducting an examination, I could distinctly appreciate the inward current of blood from the auricle to the ventricle, producing the first sound by suddenly distending this latter cavity. The apex of the heart striking against the walls of the chest in the neighborhood of the fifth and sixth ribs, communicated to the ear at this moment a shock (the heart's impulse.) Immediately followed the second sound, produced by the onward current of blood through the aortic opening, propelled by the contraction of the ventricles. I observed, moreover, that the first sound did not exceed the space in which the impulse was felt, but that the second sound was audible in nearly the whole extent of the chest, which would tend to strengthen the theory I have advanced, inasmuch as the sound produced by the diastole of the ventricle would be circumscribed, whereas that produced by the systole would be diffused.

Whilst my attention was directed to this subject, a case came under my notice in which a *bruit de râpe* was distinctly heard in the second sound of the heart, and over a considerable portion of the chest.

I diagnosed ossification of the aortic semilunar valves and arch of the aorta, believing that during the production of the second sound (systole) the current of blood was passing in this direction. The patient died shortly afterwards, and a post-mortem examination of the body disclosed the correctness of my opinion.—*London Lancet.*

